# Acid and Sulphate Attacks on a Rubberized Engineered Cementitious Composite Containing Graphene Oxide

**DOI:** 10.3390/ma13143125

**Published:** 2020-07-13

**Authors:** Lavaniyah Sabapathy, Bashar S. Mohammed, Amin Al-Fakih, Mubarak Mohammed A Wahab, M. S. Liew, Y. H. Mugahed Amran

**Affiliations:** 1 Civil and Environmental Engineering Department, Universiti Teknologi PETRONAS, Bandar Seri Iskandar 32610, Malaysia; uitm1314@gmail.com (L.S.); amin.ali_g03663@utp.edu.my (A.A.-F.); mubarakwahab@utp.edu.my (M.M.A.W.); shahir_liew@utp.edu.my (M.S.L.); 2 Department of Civil Engineering, College of Engineering, Prince Sattam Bin Abdulaziz University, Alkharj 11942, Saudi Arabia; m.amran@psau.edu.sa; 3 Department of Civil Engineering, Faculty of Engineering and IT, Amran University, Quhal, Amran 9677, Yemen

**Keywords:** crumb rubber (CR), graphene oxide (GO), response surface methodology (RSM), acid attack, sulphate attack, engineered cementitious composite (ECC)

## Abstract

The objective of this research was to determine the durability of an engineered cementitious composite (ECC) incorporating crumb rubber (CR) and graphene oxide (GO) with respect to resistance to acid and sulphate attacks. To obtain the mix designs used for this study, response surface methodology (RSM) was utilized, which yielded the composition of 13 mixes containing two variables (crumb rubber and graphene oxide). The crumb rubber had a percentage range of 0–10%, whereas the graphene oxide was tested in the range of 0.01–0.05% by volume. Three types of laboratory tests were used in this study, namely a compressive test, an acid attack test to study its durability against an acidic environment, and a sulphate attack test to examine the length change while exposed to a sulphate solution. Response surface methodology helped develop predictive responsive models and multiple objectives that aided in the optimization of results obtained from the experiments. Furthermore, a rubberized engineered cementitious composite incorporating graphene oxide yielded better chemical attack results compared to those of a normal rubberized engineered cementitious composite. In conclusion, nano-graphene in the form of graphene oxide has the ability to enhance the properties and overcome the limitations of crumb rubber incorporated into an engineered cementitious composite. The optimal mix was attained with 10% crumb rubber and 0.01 graphene oxide that achieved 43.6 MPa compressive strength, 29.4% weight loss, and 2.19% expansion. The addition of GO enhances the performance of rubberized ECC, contributing to less weight loss due to the deterioration of acidic media on the ECC. It also contributes to better resistance to changes in the length of the rubberized ECC samples.

## 1. Introduction

An engineered cementitious composite (ECC) is a type of high-performance fiber-reinforced cementitious composite (HPFRCC) with a unique property of high ductility with medium fiber content. ECCs are built to combine strong mechanical, physical, and toughness qualities even under normal or harsh conditions by using micromechanics-dependent theoretical methods [1,2]. An ECC is a composite material developed to allow the concrete industry to maximize the effective use of products, minimize waste, deliver economic and environmental benefits, and induce structural durability [3,4]. An ECC also shows high resilience to cracks, good ductility, and the ability to control crack depth, rendering it the ideal composite to improve the durability of civil infrastructures [5]. This is because ECCs are able to form steady and multiple microcracks that considerably improve its durability in the aspects of tensile strength and ductility compared to other forms of concrete [6]. An ECC has an efficiency of around 3–5% (1.03–1.05 times) more than the strength of conventional concrete, with respect to high tensile strength [7]. Studies have shown that its compressive strength varies from 20 to 95 MPa, its tensile strength ranges from 4 to 12 MPa, and its compressive strain is between 0.4% and 0.65% [8].

Although an ECC has a number of superior qualities as compared to those of conventional concrete, there are also some drawbacks that come with using this technology. First, the drying shrinkage of the ECC matrix is lower, leading to eigenstress in the composite when restrained compared to that of normal concretes. Next, regarding compression behavior, due to the absence of coarse aggregates in the ECC the elastic modulus will be lesser than that of conventional concrete as well, resulting in more strain when it attains its compressive strength [9]. When exposed to elevated temperatures, the performance of the ECC degrades as well. Fire is one of the most dangerous structural threats, as high temperatures contribute to physical and chemical modifications that weaken the ECC’s mechanical qualities, such as strength and elasticity modulus [10]. This is because of the physical–chemical changes of the cement paste and the aggregate, the alteration of pores, and the thermal incompatibility between the aggregate and the concrete paste causing internal microcracking. 

The incorporation of crumb rubber (CR) into an ECC was observed to reduce the effects of explosion and spalling because, when rubber is melted up to 170 °C, it allows space for the escape of water vapor and helps relieve pore strain. This in return decreases the destruction on the concrete structure [11]. Hernández-Olivares and Barluenga [12] indicated that CR was added to lower the danger of explosive spalling at extreme temperatures. In addition, crumb rubber has a lower specific gravity, ranging from 0.51 to 1.2, a bulk density ranging from 524 kg/m^3^ to 1273 kg/m^3^, as well as lower water absorption, strength, and stiffness compared to fine aggregates [13]. The adverse consequence of rubberized concrete is a reduction in strengths due to the hydrophobic action of the particles of crumb rubber which repels water and causes air pitfalls to the surface [14]. The cumulative findings of studies revealed a significant decline in strength and stiffness properties of concrete after the application of tire rubber cement. In fact, increasing the density of concrete will also decrease as drying shrinkage is decreased [5].

The use of rubberized aggregates in eco-friendly cementitious materials improved workability, deflection capacity, cracking behavior, impact energy, and acoustic properties [15,16,17,18]. In addition, concretes prepared with rubber aggregates were lightweight compositions with from 2% to 13% lower density than that of the control mixtures [16,19]. However, CR enhances dynamic resistance and durability but posts a negative impact on compressive strength, water absorptivity, and workability [20,21,22,23]. Hesami et al. [24] showed that the ECC’s 28-day compressive strength reduces and the lower adhesion between the paste and CR is attributed to it. Aslani [25] defined CR as voids that result in a poor linkage force between CR and the fresh paste surrounding the degenerated interfacial transition zones (ITZs). Because of its water repelling nature, CR tends to cause air voids to be entrapped and increases the number of ITZs in the cement mixture.

Previous investigations have concluded that the use of crumb rubber (CR) in an ECC harms the mechanical and durability properties due to the hydrophobic behavior of CR, which repels water around its surface [5]. Due to CR’s hydrophobic nature and air entrapment, the bonding between the cementitious materials’ matrices and CR becomes weak and less dense and thus the quality of the ECC made with CR is inferior compared to that of the normal ECC [26]. The resistance to chemical ingress in the ECC may be comparatively low when it is incorporated with CR as the residual mortar contains old ettringite and calcium monosulfoaluminate cement hydrates. Gesoğlu and Güneyisi [27] reported a progressive increase in the chloride ion penetration on the partial replacement of coarse aggregate and fine aggregate by crumb rubber and rubber chips, respectively. Sofi [28] concluded that rubberized concrete is highly resistant to aggressive environments and can be implemented in areas where there are chances of acid attack. Yung, et al. [29] investigated the durability properties of self-compacting concrete containing waste tire rubber, which indicated that the anti-sulfate corrosion was improved with the increase of rubber content from 5% to 20% of the volume ratio.

Graphene oxide (GO) (C140.H40.O20) [30] has been considered one of the most superior graphene derivatives for cementitious composites, because of its significant bonding to various oxygen functional groups and thus exhibiting higher reactivity with cement due to its large surface area [31,32]. The presence of hydrophilic functional groups in GO implies that the composites are still best dispersed [9]. Graphene is an excellent nanofiller, but is not quite durable and is expensive to modify cement products.

Previous literature has suggested that GO–cement composites had significantly higher compressive and bending forces (over 100% depending on the formulation used) in the same mixture proportions than those of conventional cement composites [33,34]. This is because the ECC’s durability is enhanced by including GO, thereby optimizing the composition of the micropore, avoiding the initiation and proliferation of microcracks at the beginning, enhancing transport properties (water permeability, gas permeability, and tolerance to chloride penetration), and increasing the freezing and tanning process tolerance [9]. The overall porosity of cement composites of 1 wt% GO was also found to be decreased from 25.21% to 10.61% [35]. For these reasons, GO is intended to help increase the product density of calcium silica hydrate (C-S-H), reduce the porosity of the microstructure, and stabilize the composites [36]. Research has found that specimens incorporating GO and at elevated temperature displayed increased specimen weight and dimensional stability and tolerance to spalling [9].

Concrete is prone to certain types of chemical attacks that can severely impact its mechanical and physical properties. The penetration of different chemicals into concrete members may lead to failure such as strength loss, cracking, and corrosion of the cement paste of concrete [37]. In the case of acid attacks on concrete, the degradation of concrete members exposed to aggressive sulfuric acid environments is a crucial durability issue that affects the life cycle performance and maintenance costs of vital civil infrastructure. Acids present in the environmental groundwater or in chemical wastewater reduce strength and corrode concrete [38]. Moreover, concrete structures in industrial zones are susceptible to deterioration due to acid rain, of which sulfuric acid is a chief component. Portland cement concrete hydration items are alkaline and have a pH rating of 12 to 13.5 [39]. For sulphate attacks on concrete (sodium sulphate), there are two types, namely external and internal. An external sulphate attack is the penetration of salt-bearing solutions into the concrete and forms ettringite [40]. As for the internal attack, it occurs when the mixing components of concrete are exposed to sulphate. The gypsum present in the concrete reacts with monosulfates to form ettringite. The development of ettringitis is believed to be the primary source of the expansion and destruction of sulphate attack concrete systems [41]. Furthermore, a chemical sulphate attack occurs when sulphate penetrates the concrete and reacts with the hydration products of the concrete. It occupies a more noteworthy volume, causing expansion inside the cement mix and bond, which at that point creates an interior and concentrated tensile stresses in hardened concrete [42]. The inclusion of GO presents an effective solution as compared to traditional fibers as it constructs modifications and achieves better performance at the nanoscale due to its higher specific surface area and availability of larger functional groups [43]. The addition of GO provides planes for the reaction of cement hydration products and the formation of strong covalent bonds [44]. As a result, it enhances the structural interface and strengthens the performance of cementitious composites [9]. 

Graphene oxide (GO) has been used to improve the hydration of cementitious material to make the concrete denser and more durable [45]. The oxygenated functionalities attached make GO a highly dispersible reinforcing agent in cement matrix compared to other carbon-based nanomaterials such as carbon nanotubes, carbon nanofibers, etc., which easily agglomerates in the cement-based composites [46,47]. Mohammed et al. [48] confirmed that the GO inclusion led to improvement in porosity of GO-reinforced cement-based composites, thus the resistance to chloride ion penetration increases and the sorptivity value reduces with percentage increments of GO. Previous studies have concluded that GO incorporation accelerates the hydration in cement. This may be attributed to the oxygenated functional groups attached to GO nanosheets, which makes them more approachable to cement particles, further boosting the reaction of cement with water by acting as nuclei for the cement phases [49]. Hence, GO addition in concrete seems a promising nanomaterial for enhancing cement-based composites. Zhao, et al. [50] reported that graphene oxide (GO) is a derivative of graphene, which can be viewed as a layer of graphene with grafted oxygen functional groups. These active functional groups prefer to participate in chemical or physical interactions, which can improve the interfacial bonding with the host materials. Lin, Wei and Hu [44] concluded that the addition of GO provides planes for the reaction of cement hydration products and the formation of strong covalent bonds. Furthermore, Zheng, Han, Cui, Yu and Ou [9] found that GO enhances the structural interface and strengthens the performance of cementitious composites. 

Therefore, the inclusion of GO has the potential of minimizing the challenges related to the full-scale utilization of an ECC and the adverse effects of CR in an ECC by improving the weak bond between the CR and the cementitious materials’ matrices. The main aim of the current study was to determine the durability of an ECC incorporating crumb rubber and GO with respect to resistance to acid and sulphate attacks.

## 2. Materials and Experimental Procedure

### 2.1. Materials and Properties

**Figure 1:**
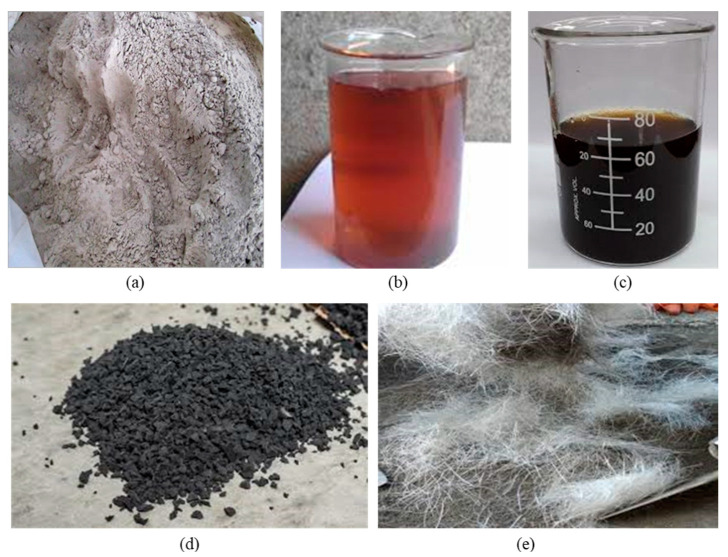
Materials used: (**a**) class F fly ash; (**b**) superplastisizer (Sika Viscocrete-2044); (**c**) graphene oxide; (**d**) crumb rubber; and (**e**) PVA.

**Figure 2:**
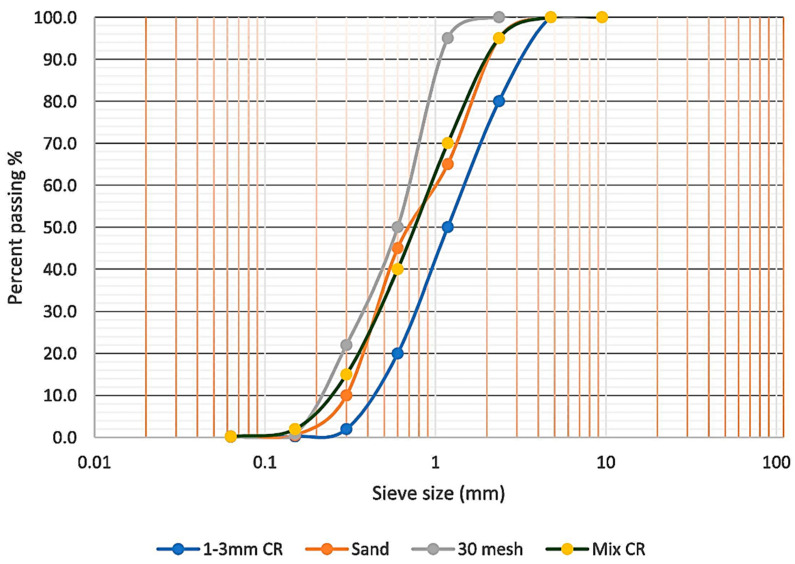
Grading curve of fine sand and crumb rubber.

In this research, the materials used in the fabrication of a rubberized ECC containing nanographene are shown in Figure 1 as follows. (1) Ordinary Portland cement (OPC, Tasek Concrete, Selangor, Malaysia) Type I in line with the specifications of ASTM C150 [51]. (2) Class F fly ash (FA, Manjung Power Plant, Perak, Malaysia) in accordance with the requirement specifications prescribed in ASTM C618-17 [52] with a density of 1300 kg/m^3^ and an amount of (Al_2_O_3_ + Fe_2_O_3_ + SiO_2_) 82.12% and below 6% of loss on ignition. FA was used in the mixture to reduce the cost of the material, and it behaved like an intense water-reducing substance. FA is a byproduct of pulverized coal being burned in thermal electric generation plants and is waste material that has pozzolanic properties classified as cement-replacing materials. The chemical contents of OPC and FA are presented in Table 1. (3) Local washed river sand was used in the mixes conforming to ASTM C33-M16 [53]. The sizes of 0.3–1.18 mm and the sand/cementitious ratio of 0.36 were utilized to maintain the adequate stiffness and volume stability and also to obtain the better fresh and hardened properties of rubberized ECC containing GO. (4) CR particles were varied from 0% to 10% and used as a partial replacement of sand by volume, combining sieve size of 30 mesh and sieve size of 1 to 3 mm in the appropriate mixed proportions of 60% and 40%, respectively [54]. In order to reach a trend similar to that of sand particles, where the sand is replaced with the crumb rubber, the final gradation of crumb rubber contained 60% of passing size #30 mesh and 40% of passing size 1–3 mm. The specific gravity of crumb rubber is 0.95, replacing the amount of fine aggregates by volume percentage. The sieve analysis of the fine aggregate and crumb rubber is shown in Figure 2 (5) Polyvinyl alcohol (PVA, Kuraray, Chiyoda-ku, Tokyo 100-8115, Japan) fibers (Table 2) were added to cementitious composites with a fixed volume of 2% to achieve uniform dispersion and workability and to respect the principles of micromechanics requirements in order to improve ductility and high strain in the cementitious matrix. (6) Graphene oxide (GO, Graphenea, San Sebastián, Spain) having a concentration of 4 mg/mL was used and ranged from 0.01% to 0.05%. The physical properties of GO and its elemental analysis are shown in Table 3 and Table 4. An aqueous solution of superplasticizer known as modified polycarboxylate-based (HRWR) “Sika Viscocrete-2044” was used to adjust the mixtures to obtain the desired flowability. Sika Viscocrete-2044 (Sika Kimia, Negeri Sembilan D.K., Malaysia) is a polycarboxylate superplasticizer (SP) in liquid form with a 6.2 pH value and a 1.08 specific gravity, and there is absence of chloride ion content. Water that is suitable for drinking is usually considered acceptable for mixing the concrete. In this study, the water-to-cement ratio was set to 0.215.

### 2.2. Response Surface Methodology (RSM)

In the current study, experiments were conducted according to a rotatable central composite design (CCD) using response surface methodology (RSM) with 2 independent (CR and GO) variables (N). The design consisted of a total of 13 mixes (2 ^N^ + 2 N + 5, where N = number of independent factors) with 5 central points. This method has been followed by many researchers [4,59,60,61,62,63]. Therefore, 13 mix designs with varying percentages of CR and GO were obtained, as shown in Table 5. The final products produced from these mixes were used for various experimental tests including compressive strength, acid attack test, and sulphate attack test to determine their performance. The range of CR as a partial replacement of fine aggregates was varied from 0% to 10% by volume, while the introduction of GO into the matrix was 0.05%, 0.03% and 0.01%.

Another role played by RSM is in the analysis of the results obtained. The analysis of variance (ANOVA) was used to display the reliable declaration of estimations of the various tests done for this research. By using RSM, the response optimization of output variables (compressive strength, acid attack, and sulphate attack) was estimated using its input variables (CR and GO), which influenced the results obtained.

### 2.3. Testing Setup

A total of 13 mixtures were prepared, cast, and left for curing for 28 days. For each mix, 3 experimental tests were carried out including compressive strength test, acid attack (weight loss) test, and sulphate attack (length change) test. For each test, 3 molds were prepared to obtain the average results. The thirteen mixtures with three different proportions of GO (0.05%, 0.03%, and 0.01%) by volume and three levels of crumb rubber replacement (0%, 5%, and 10%) to fine aggregate by volume were considered, as shown in Table 5.

**Figure 3:**
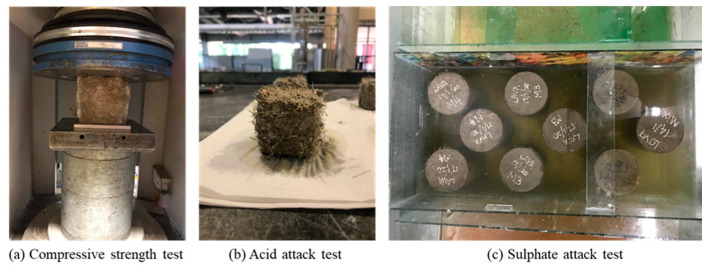
Samples and testing setup: (**a**) Compressive strength test; (**b**) Acid attack test; (**c**) Sulphate attack test.

A compressive strength test was carried out according to BS 1881: Part 116:1983 [64]. Cubes of 50 mm × 50 mm × 50 mm were prepared and cast, and then left in the curing tank for 28 days. Once the curing period was completed, the cubes were removed from the tanks and air-dried and underwent a compressive test using the UTM machine, as shown in Figure 3a. For the acid attack test, 3 cubes per mix with the dimensions of 50 mm × 50 mm × 50 mm (Figure 3b) were prepared according to ASTM C642 [65] in order to obtain normal weight concrete, approximately 800 g. After 24 h, samples were demolded and kept in ambient atmosphere for 2 days. Afterward, samples were weighed for initial weight before immersion in an acid solution. Next, samples were carefully immersed in a 5% sulfuric acid (H_2_SO_4_) solution for 28 days. After completing the immersion period, samples were taken out and kept in ambient atmosphere for 2 days to dry and achieve constant weight. Samples were then weighed for final weight. The loss in weight and percentage weight loss were then calculated.

The sulphate attack test (Figure 3c) was conducted following the guidelines according to ASTM C1012 [66]. Cylinders having dimensions of 50 mm diameter × 200 mm height were prepared in order to facilitate the measurement of the expansion due to the sulfate solution exposure. The samples were cast and air-cured in molds for 24 h. After 24 h, the samples were demolded and the initial vertical length of the cylinders was recorded. Next, the cylinders were immersed in a 10% sodium sulfate solution for 28 days. At the end of the 28-day immersion period, the cylinders were removed from the solution and the final lengths were recorded.

## 3. Results and Discussion 

### 3.1. Compressive Results

**Figure 4:**
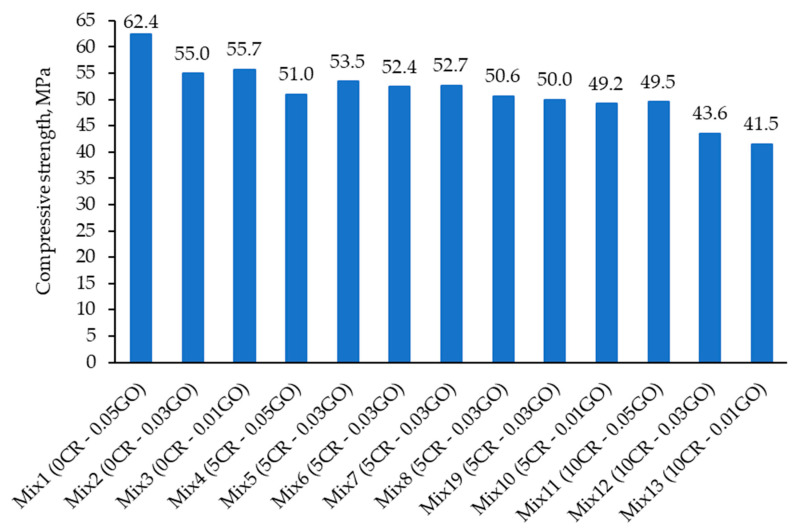
Compressive strength test results of the mixtures at 28 days.

Figure 4 shows the compressive strength test results of the 13 mixes at 28 days of rubberized ECC incorporating GO. The mix with the highest compressive strength was Mix 1, with a strength of 62.4 MPa. It had the lowest percentage of crumb rubber (0% CR) and the highest percentage of graphene oxide (0.05% GO). On the other hand, the mix with the lowest compressive strength was Mix 13, having the highest percentage of CR (10%) but the lowest percentage of GO (0%) as compared to Mix 1. 

**Figure 5:**
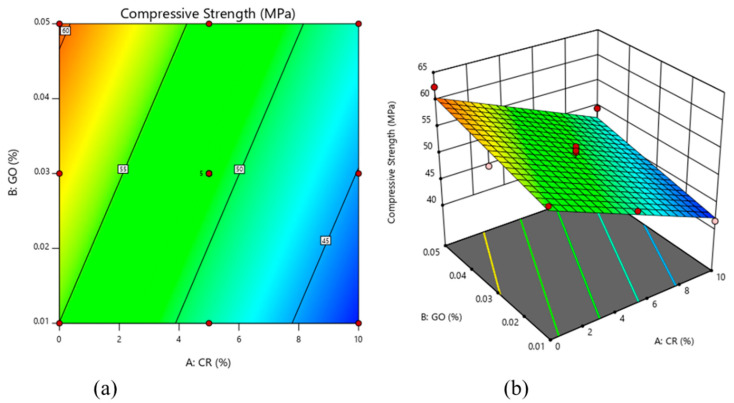
Compressive strength of rubberized ECC with GO: (**a**) 2D contour plot and (**b**) 3D surface response.

Figure 5 shows that the compressive strength decreased gradually as the CR percentage increased, whereas the increment of GO percentage led to an increase in the compressive strength. This finding is in line with the previous studies that confirmed the negative effect of added CR to the compressive strength of the ECC [5,26].

**Figure 6:**
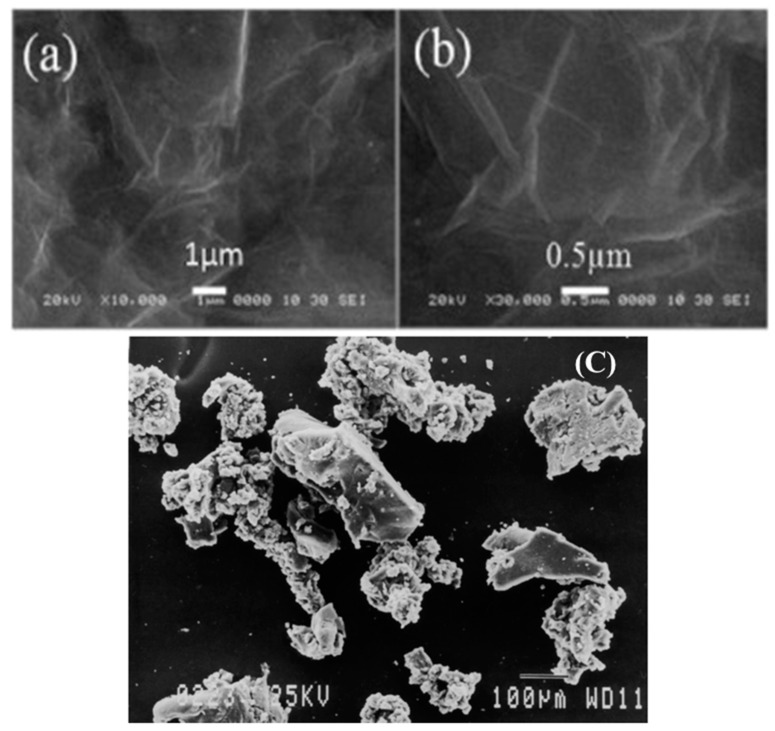
The scanning electron microscopy (SEM) of (**a**,**b**) GO [67] and (**c**) CR particles [68].

To explain the results obtained, the two variables (CR and GO) have an opposing effect on the compressive strength of the ECC. Meanwhile, CR has the tendency to repel water due to its hydrophobic nature and to entrap air, increasing the number of ITZs, and causes poor bonding between the particles, as shown in Figure 6c. On the contrary, GO has the potential to enhance the strength of the ECC mix matrix due to its hydrophilic properties, as shown in Figure 6a,b. It is dispersed well throughout the mix and aids in the increase of the compressive strength of the concrete. Therefore, GO is proven to enhance the previously occurring limitations of CR when incorporated into the ECC matrix.

### 3.2. Acid Attack Test Results

**Figure 7:**
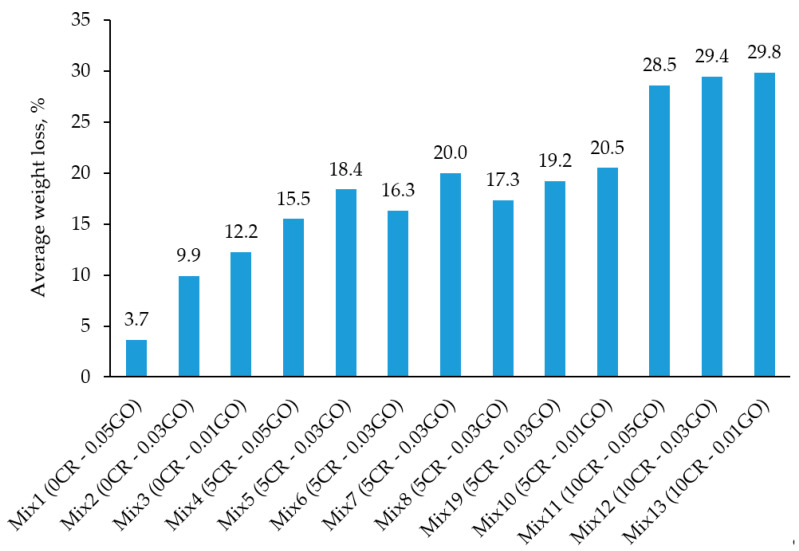
Acid attack test results at 28 days.

Figure 7 shows the results of the acid attack test of the 13 mixes of the rubberized ECC containing GO. The results were obtained by calculating the average percentage weight loss of the mixes after the samples were immersed in a 5% suspension of sulfuric acid (H_2_SO_4_) for 28 days. From the results, it can be deduced that the lowest average weight loss (3.7%) was reported in Mix 1, having the highest percentage of GO (0.05%) and lowest percentage of CR (0%). On the other hand, Mix 13 was reported to have the highest weight loss percentage of 29.8%. Mix 13 had the highest CR (10%) and the lowest GO percentage content of 0.01%. 

**Figure 8:**
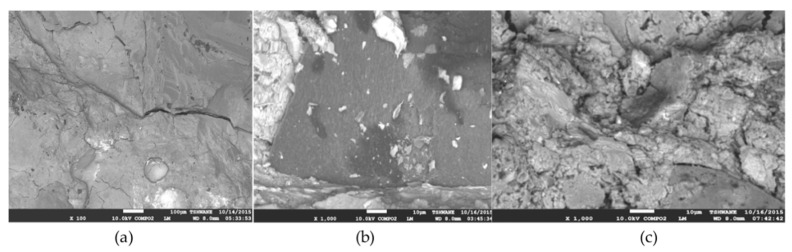
SEM images of rubberized ECC containing (**a**) 0% CR, (**b**) 5% CR, and (**c**) 10% CR.

The findings from this test can be explained by observing the variation in the content of the two variables present, namely CR and GO. The CR particles present in the rubberized ECC caused microcracking in the samples, causing more penetration of acidic media. However, in the rubberized ECC with no CR or a smaller amount of CR, fewer cracks were developed and the constituent materials were less easily separated, causing a lower percentage of weight loss due to deterioration, as shown in Figure 8. The GO presented in the mix further aided in increasing the durability performance of the rubberized ECC when subjected to harsh environments, such as acidic environments. The mixes with lower GO had higher percentages of weight loss due to the deterioration of rubberized ECC, while those with higher GO were more durable. When comparing mixes having the same ratio of CR content but different values of GO, it can be seen that the higher GO content produced better results in terms of weight loss. A similar trend was observed by Sofi [28] where he found that, at a water/cement ratio 0.4, 0.45 and 0.5, a greater amount of weight loss was observed in the control mix specimens, and it was found that it decreased as the amount of crumb rubber was increased in the concrete. It means that the control mix specimens have recorded maximum loss in weight and the specimens with 20% crumb rubber have recorded the least loss in weight. This is because the crumb rubber particles present in the rubberized concrete were holding the constituent particles of the concrete from breaking away by preventing the formation of cracks and material separation.

**Figure 9:**
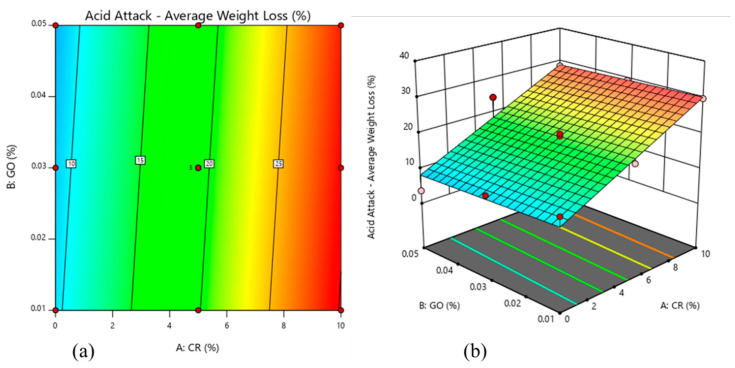
Acid attack of rubberized ECC with GO: (**a**) 2D contour plot and (**b**) 3D surface response.

Figure 9 shows that the weight loss of rubberized ECC incorporating GO decreased with an increase of the CR replacement, while the GO has shown to have an optimal effect on the weight loss at around 0.03%/0.01%. Both CR and GO play important roles in improving the durability of ECC in harsh environments. Thus, it is clear that the rubberized ECC incorporating GO is satisfactorily resistant to the aggressive environments.

### 3.3. Sulphate Attack Test Results 

**Figure 10:**
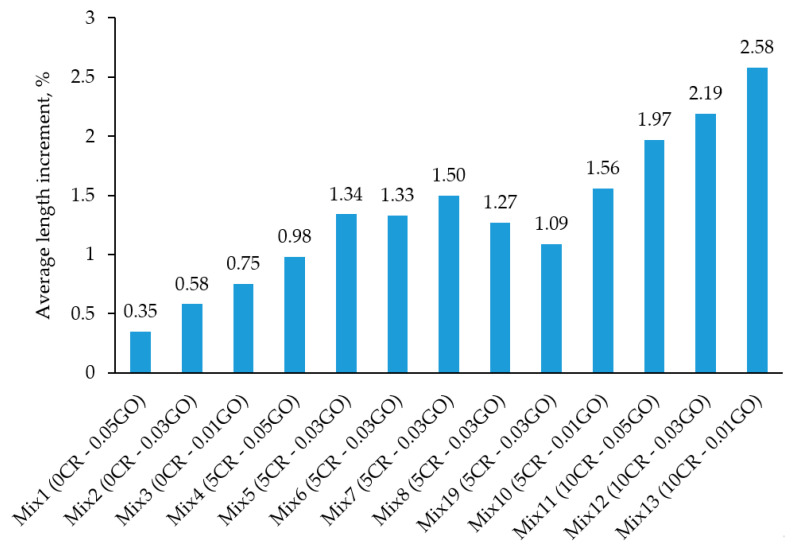
Sulphate attack test results of rubberized ECC with GO after 28 days.

Figure 10 shows the results of the sulphate attack test that measured, as an average, the change in length of cylinders when immersed in a suspension of 10% sodium sulphate solution (Na_2_SO_4_). The average increment in length was calculated by comparing the average lengths of cylinder samples of each mix before and after 28 days of immersion in sulphate media. The percentage increase was then calculated.

From the results, it can be observed that Mix 1 had the lowest average percentage in length change at 0.35%. It had no CR in the ECC matrix but had the highest content of GO (0.05%). The highest reported length change was observed in Mix 13, which had a percentage of 2.58% in length change and had the lowest GO content (0.01%). A similar trend could be observed for the concrete with a replacement of mixture by crumb rubber exceeding 5% that could not significantly enhance sulfate resistance [69].

**Figure 11:**
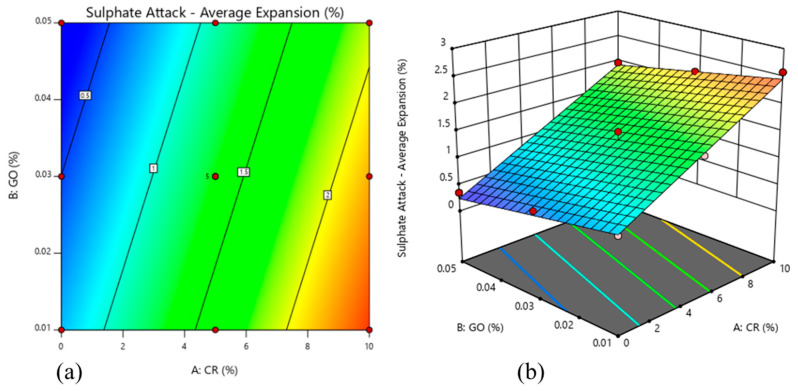
Sulphate attack of rubberized ECC with GO: (**a**) 2D contour plot and (**b**) 3D surface response.

Figure 11 shows the 2D contour plot and 3D surface response, which represent the interaction effect of CR replacement and the addition of GO on the change of length of rubberized ECC incorporating GO. As seen by the 3D surface reaction, expansion increased with the increase in CR replacement, whereas the GO percentage decreased the expansion of rubberized ECC.

The findings from these results can be explained by the presence and absence of CR at different percentages and also the percentage of GO presented from the range of 0.01% to 0.05% in different mixes. An increase in length change in mixes containing a higher CR content is due to the occurrence of microvoids around the surface of the specimens, which have enabled more absorption of immersed media. The more CR present in the mix, the higher the number of microvoids present (as shown in Figure 8), hence resulting in more expansion. This is in line with the study that reported that the depth of chloride penetration of the concrete mixes with crumb rubber above 5% was higher than that of the control mix. The top layer of the concrete specimens with 0% crumb rubber was completely removed (100%) by the action of sulfuric acid. In the case of the mix with 20% crumb rubber, less than 100% top surface was attacked by acid [28].

The GO content, on the other hand, had beneficial impacts at reducing the length change of concrete due to its resistivity against penetration of the sulphate solution media. The mechanism of resistivity is that, during the dissolution and setting stages, the electrical resistivity of rubberized ECC incorporating GO generally increased due to the decrease of free water, which was caused by the high adsorption capacity of GO with large surface areas. With the process of hydration, the increase in solid hydration products and the decrease of porosity tended to block the conduction path of ions in rubberized ECC incorporating GO paste, thus increasing the resistivity.

A higher GO content is able to reduce the expansion effects of concrete due to the penetration of sulphate suspension. The increase in expansion was also due to the growth of ettringite-filled holes and cracks on the surface of the cement composite [40,70]. Moreover, the formation of ettringites employs a larger area, being responsible for the expansion and cracking behavior of ECC [71].

### 3.4. Validation and ANOVA Analysis of Rubberized ECC Containing GO

RSM is a collection of mathematical and statistical techniques for empirical model building. Its main purpose is to explore the relationships between several independent variables and one or more dependent variables and then to optimize responses based on the results [72]. In other words, the output variables are influenced by the input variables. Table 6 shows the mix design obtained using RSM and the corresponding responses for three different dependent variables.

The range of CR as a partial replacement of fine aggregates was varied from 0% to 10% by volume, while the introduction of GO into the matrix ranged from 0.05% to 0.01%. It was observed that mixes 5–9 had similar percentages of both CR and GO. These five replicates are the central point that the software (RSM) used to improve the precision of the experiment against any likely errors [40,62,63,70]. This is because, during optimization by RSM, the CR percentage of 5% and the GO percentage of 0.03% are considered as the central point; therefore, repetition is required for the accuracy of data and results. The correlations between the factors CR and GO and the responses (compressive strength, acid attack, and sulphate attack) were given in the developed model equations shown below, where Equation (1) is for compressive strength, Equation (2) is for weight loss. and Equation (3) is for expansion (change in length):

(1) Compressive Strength=53.60776 − 1.28267 × CR+137.08333 × GO

(2) Average Weight Loss=9.83609+2.06567 × CR − 31.91667 × GO

(3) Average Expansion=0.899551+0.168667 × CR − 13.25000 × GO

The analysis of variance (ANOVA) displayed a reliable declaration for the estimations of responses 1, 2, and 3. R^2^ shows the accuracy of the predicted value with the experimental value or the actual value. The coefficient of determination is one of the criteria used to evaluate how close the data are to the fitted regression line. For each response, the predicted R^2^ and the adjusted R^2^ have a difference of less than 0.2. Table 7 also shows that the *p*-value for all responses is less than 0.05. This is an indication that the models are significant, as a significance level of 0.05 or more indicates 5% and a higher risk of concluding that the model explains variation in the response when the model does not. The F-values for all three responses are also significant, indicating that there is only a 0.01% chance that large F-values obtained are due to noise.

**Figure 12:**
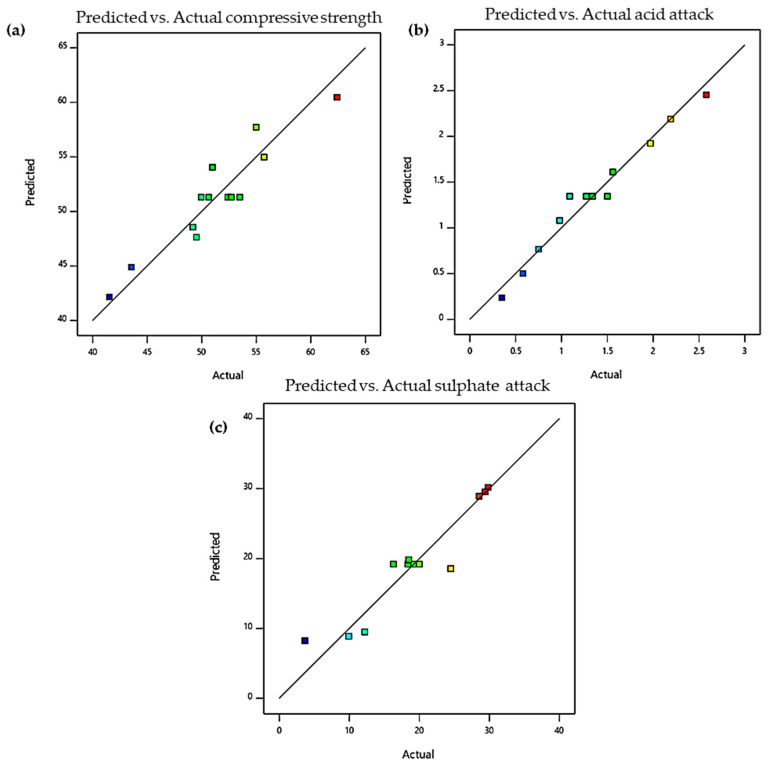
Normality plot of residuals for (**a**) compressive strength, (**b**) acid attack, and (**c**) sulphate attack.

To further confirm the adequacy of the models, the prediction versus actual values and their corresponding normality plots of residuals of the responses are presented in Figure 12. The normal percentage and externally studentized residual plots for the responses showed good agreement since all the points fall along the straight line in all the responses. This confirms that the models can be conveniently used to traverse the design space. The latter proves that the model’s fairness as residuals (the difference between experimental data and fitted values predicted by the model) are randomly distributed around zero. All the normality plots show that some responses differ from their predicted values. However, as long as it is within the red control limits, it is still acceptable. The models were selected based on the sum of squares of the higher order polynomial, where they are not aliased, and the model terms were statistically significant.

## 4. Conclusions

Based on the results obtained, the following conclusions can be drawn:

- Crumb rubber incorporated into ECC has its limitations in terms of strength and durability. The hydrophobic nature of crumb rubber, which causes it to repel water and trap air in its voids, leads to the reduction of the strength of the ECC.
- The addition of nanographene in the form of GO has the ability to overcome the limitations of the rubberized ECC.
- The higher CR content in the rubberized ECC makes the concrete more susceptible to acid attacks due to the multiple microcracking behaviors caused by CR. Meanwhile, CR has the tendency to repel water due to its hydrophobic nature and to entrap air, increasing the ITZs and causing poor bonding between the particles.
- The addition of GO helps enhance the performance of rubberized ECC, aiding in less weight loss due to the deterioration of acidic media on the ECC.
- The addition of GO in the matrix contributes to better resistance to changes in the length of the rubberized ECC samples.
- RSM enables the targeted performance of the rubberized ECC incorporating GO to be achieved with reasonable precision across several mixtures. The results showed that the quadratic model can predict the properties of new mixtures with reasonable accuracy by comparing experimental results and those that the statistical data predicted.

## Figures and Tables

**Table 1:** Chemical constituent of (OPC) and FA [55,56].

Constituents (%)	FA,%	OPC,%
SiO_2_	64.69	25.21
Al_2_O_3_	18.89	4.59
Fe_2_O_3_	4.9	2.99
CaO	5.98	62.85
MgO	1.99	1.7
Na_2_O	2.41	0.98
K_2_O	1.53	0.78
Loss on ignition	1.87	2.02
Specific gravity	2.3	3.15

**Table 2:** Properties of polyvinyl alcohol (PVA) fiber [57].

Type	Specific Gravity	Density (g/cm^3^)	Fiber Dia. (μm)	Fiber Length (mm)	MOE (GPa)	Tensile Strength (MPa)	Aspect Ratio (l/d)
PVA	1.3	1.31	40	12	41	1600	462

**Table 3:** Physical properties of graphene oxide [58].

Form	Particle Size, μm	Odor	Color	Concentration (wt%)	Dispersibility	pH (4 mg/L Dispersion)
Slurry	6 to 33	Odorless	Dark brown	2.5	Polar solvents	1.8 to 2.0

**Table 4:** Elemental analysis of graphene oxide [58].

Carbon	Nitrogen	Hydrogen	Oxygen	Sulphur
49–56%	0–1%	0–1%	41–50%	2–4%

**Table 5:** Mixture proportions with amount of raw materials of rubberized engineered cementitious composite (ECC) containing GO.

Mix	Cement kg/m^3^	FA kg/m^3^	Sand kg/m^3^	CR		Water kg/m^3^	SP kg/m^3^	PVA		GO	
				%	g			%	kg/m^3^	%	g
1	570	684	451.44	0	0.00	269.61	2.51	2.0	25.08	0.05	9.59
2	570	684	451.44	0	0.00	269.61	2.51	2.0	25.08	0.03	5.76
3	570	684	451.44	0	0.00	269.61	2.51	2.0	25.08	0.01	1.92
4	570	684	451.44	5	759.83	269.61	2.51	2.0	25.08	0.05	9.59
5	570	684	451.44	5	759.83	269.61	2.51	2.0	25.08	0.03	5.76
6	570	684	451.44	5	759.83	269.61	2.51	2.0	25.08	0.03	5.76
7	570	684	451.44	5	759.83	269.61	2.51	2.0	25.08	0.03	5.76
8	570	684	451.44	5	759.83	269.61	2.51	2.0	25.08	0.03	5.76
9	570	684	451.44	5	759.83	269.61	2.51	2.0	25.08	0.03	5.76
10	570	684	451.44	5	759.83	269.61	2.51	2.0	25.08	0.01	1.92
11	570	684	451.44	10	1519.67	269.61	2.51	2.0	25.08	0.05	9.59
12	570	684	451.44	10	1519.67	269.61	2.51	2.0	25.08	0.03	5.76
13	570	684	451.44	10	1519.67	269.61	2.51	2.0	25.08	0.01	1.92

**Table 6:** Mix combinations and response results.

Mix	Variables		Responses		
	CR (%)	GO (%)	Compressive Strength, MPa (COV,%)	Acid Attack—Average Weight Loss (COV,%)	Sulphate Attack—Average Expansion (COV,%)
1	0	0.05	62.39 (5.26)	3.66 (4.33)	0.35 (5.01)
2	0	0.03	54.98 (3.15)	9.92 (4.59)	0.58 (3.25)
3	0	0.01	55.71 (6.51)	12.21 (6.02)	0.75 (6.57)
4	5	0.05	50.98 (5.55)	15.49 (3.21)	0.98 (4.25)
5	5	0.03	53.48 (4.93)	18.38 (7.77)	1.34 (6.57)
6	5	0.03	52.38 (7.50)	16.29 (3.92)	1.33 (5.69)
7	5	0.03	52.71 (2.67)	20.00 (3.42)	1.50 (6.33)
8	5	0.03	50.63 (4.02)	17.32 (2.89)	1.27 (4.69)
9	5	0.03	49.95 (3.11)	19.17 (1.54)	1.09 (5.48)
10	5	0.01	49.18 (1.95)	20.49 (3.52)	1.56 3.61)
11	10	0.05	49.51 (6.03)	28.54 (5.21)	1.97 (4.56)
12	10	0.03	43.55 (2.68)	29.40 (4.09)	2.19 (2.15)
13	10	0.01	41.54 (2.48)	29.82 (6.95)	2.58 (7.23)

**Table 7:** ANOVA analysis.

Responses	Source	F-Value	*p*-Value	Significance
Compressive Strength	Model	39.03	<0.0001	Yes
	Lack of Fit	2.17	0.2361	No
Acid Attack	Model	42.09	<0.0001	Yes
	Lack of Fit	5.55	0.0595	No
Sulphate Attack	Model	161.78	<0.0001	Yes
	Lack of Fit	0.4397	0.8238	No
